# Eating disorder and suicide attempt

**DOI:** 10.1192/j.eurpsy.2022.1489

**Published:** 2022-09-01

**Authors:** A. Chaara, F. Laboudi, A. Ouanass

**Affiliations:** 1 Arrazi Psychiatric Hospital, Emergency Service, Salé, Morocco; 2 university hospital center, University Psychiatric Hospital Ar-razi, SALE, Morocco; 3 University Hospital Center Ibn Sina, Ar-razi Psychiatric Hospital, Salé, Morocco

**Keywords:** Suicide, eating disorder

## Abstract

**Introduction:**

The quantification of suicidal risk in specific populations is important for the adoption of prevention and risk reduction measures. This risk remains very high in patients with eating disorders compared to the general population.

**Objectives:**

The present study evaluates the prevalence of suicide among patients with eating disorders all seeking different suicidal risk factors in these patients.

**Methods:**

A retrospective study of medical records of all patients with eating disorders, diagnosed according to DSM 5 criteria, at Arrazi hospital of Salé for the past 14 years, by assessing the prevalence of suicide attempts and care.

**Results:**

In our work, all patients are female, 17 years old on average, 18 patients out of 20 have anorexia nervosa, 4 of them have had bouts of bulimia, and only one patient was admitted for management of bulimia nervosa alone. The mean age of onset was 15 years with addictive comorbidity in 5 patients.

Thirteen patients had comorbid depressive disorder, one patient was schizophrenic and six patients had borderline personality disorder.

12 patients have made suicide attempts, planned especially in a depressive environment.

**Conclusions:**

Preventive management of suicidal risk must be taken in order to improve the prognosis in this category of patients.

**Disclosure:**

No significant relationships.

